# Deep learning based approach for Behavior classification in diagnoses of Autism Spectrum Disorder using naturalistic videos

**DOI:** 10.3389/fncom.2026.1626315

**Published:** 2026-03-18

**Authors:** Usama Jabbar, Muhammad Waseem Iqbal, Alexandru Nechifor, Mohammed Abaker, Mohammed Ahmed Khairalseed, Valentin Marian Antohi, Costinela Fortea, Catalin Aurelian Stefanescu

**Affiliations:** 1 Department of Computer Science, Superior University, Lahore, Pakistan; 2 Department of Software Engineering, Superior University, Lahore, Pakistan; 3 Department of Individual Sports and Physiotherapy, Faculty of Physical Education and Sport, Dunarea de Jos University, Galaţi, Romania; 4 Applied College, King Khalid University, Muhayil, Saudi Arabia; 5 Faculty of Education, University of Gadarif, Gadarif, Sudan; 6 Department of Business Administration, Dunarea de Jos University of Galati, Galaţi, Romania; 7 Department of Finance, Accounting and Economic Theory, Transilvania University of Brasov, Brasov, Romania; 8 Department of Sports Games and Physical Education, Faculty of Physical Education and Sport, Dunarea de Jos University of Galati, Galaţi, Romania

**Keywords:** Autism Spectrum Disorder, Behavior analysis, CNN-GRU, deep learning, naturalistic video

## Abstract

Autism Spectrum Disorder (ASD) is a neurodevelopmental disorder that is marked by a lack of communication skills in social situations and repetitive and stereotypical Behaviors. The most widespread form of diagnosing ASD among children is based on psychological screening test along with monitoring of the Behavioral pattern, especially repetitive Behaviors. Some of these Behaviors include hand-flapping, head banging and spinning which are common among ASD children. In our research, we examine abnormal Behavioral patterns that may reflect ASD through the videos of children engaged in the everyday activities in the unstructured settings. A publicly available multiclass Self-Stimulatory Behavior Dataset (SSBD) is use in classify autistic Behavior. Before training the model, the dataset is thoroughly pre-processed (region-of-interest (ROI) detection and image cropping to eliminate irrelevant background objects). Moreover, information-augmenting methods are used to reduce overfitting and increase training efficiency and generalization effectiveness. In order to obtain spatiotemporal details successfully, a number of deep learning models are tested, such as studied CNN-GRU model, 3D-CNN + LSTM, MobileNet, VGG16, and EfficientNet-B7. The findings of the experiment prove that the proposed CNN-GRU model is superior to all competing methods. The model with a k-fold cross-validation provides a steady accuracy of 0.9284 ± 0.0039–0.9294 ± 0.0038, which means that the model is robust and consistent across the folds. The effectiveness of the proposed approach is additionally justified by the comparisons with state-of-the-art methods. The results show that the systems based on the action recognition can help clinicians monitor the Behavioral trends and facilitate the quick, accurate, and effective screening of ASD. The proposed approach works effectively in predicting Behavior in real-life, uncontrolled videos and shows tremendous potential for real-world clinical implementation as a decision-support tool.

## 1 Introduction

Autism is a neural disease that influences the ability of a person to communicate effectively and do social interactions. It is a nervous behavior disorder, which is accompanied by repetitive actions, impairment of social interaction, communication, and language. A combination of these symptoms is known as autism (White et al., 2009; Cabanillas-Tello and Cabanillas-Carbonell, 2020; Kamran et al., 2022). Though the exact causes behind the rising rate of autism in children are not well understood, still, many of them still need intensive support and care throughout their life even when they are treated at early stages of the condition. However, early intervention is vital to the enhancement of long-term outcomes (Bilal et al., 2022). According to population-based studies, emotional and Behavioral issues in children with ASD can range from 40 to 50 percent and are clinically significant (Totsika et al., 2011). ASD children's diagnoses, severity levels, and skill assessments have largely been achieved through conventional diagnostic techniques. There are two traditional techniques, such as the rating scale and functional assessment, that are used for diagnosis and observing the Behavior of autistic patients. The rating scale method asks a series of questions and calculates the score. If the score exceeds the threshold value, the patient has autism. In-depth observations and assessments of children's abilities in various areas, including self-stimulatory Behavior, joint attention, independent play, social interaction, and the recognition of emotions from facial expressions, are part of the functional assessment. Determining how children feel is one of the challenges we have while working with them, particularly when it comes to autistic children who have a hard time adjusting to their surroundings. Using assistive technologies and figuring out how to make the most of the usage of technology and intelligent systems to help these kids is one way to solve this challenge (Case-Smith et al., 2015; Roane, 2016). Children with autism tend to be quiet, yet they can copy specific actions from cartoons and movies. As a result, they might act dangerously or unexpectedly (Abdel Hameed et al., 2022). However, there are several restrictions when employing traditional diagnostic and functional assessment techniques. First off, interpreting the observed Behavior of an autism patient is manual and takes time. Second, a clinician's observations may not always be trustworthy or valid due to variations in professional training, experience, resource availability, and cultural backgrounds. Employing AI (artificial intelligence) approaches for early identification and neurological evaluations of ASD has been shown to have substantial advantages (Alwakeel et al., 2015). In the past era, facial analysis and computer vision-based Behavior imaging have demonstrated promising outcomes in aiding doctors in the identification of a variety of medical problems, including ASD (Kohli et al., 2022). Even though computer vision has shown many promising applications, its use in evaluating Behavior, play, imitation, life skills, posture, and gait analysis to measure the joint attention of ASD children has not yet been investigated (Su et al., 2020). Computer vision-based intelligent activity monitoring and irregularity prediction in real-time can offer a trustworthy environment of assistance for those with mental or physical impairments. The ability to recognize activity has been substantially revolutionized by combining cutting-edge data processing techniques with computer vision monitoring concepts (Klintwall and Eikeseth, 2014; Ye et al., 2013). Despite the many potential solutions that computer vision has shown in different fields, in the context of autism Behavior monitoring and classification of abnormal Behavior has not yet been investigated. The purpose of this study is to create a computer vision-based monitoring framework that can classify different Behavioral actions from naturalistic videos of children with ASD, helping the clinician make an ASD diagnosis and monitor their Behavior. We do not intend to conduct clinical diagnosis of ASD in this study, but instead, we aim at examining observable behavioral cues in unconstrained video data as positive indicators, which may help in primary screening. The suggested video-based framework will be used as a supplement to the current clinical assessment protocol, which will offer an automated assistive screening tool which may then be used to bring to attention the behavioral trends of interest to be investigated further by the respective professionals. The given approach can be especially helpful in the context of large-scale screening or resource-intensive settings, where the number of trained clinicians is scarce. The ultimate diagnosis and interpretation must be left to a competent healthcare system and a suggested method is to be considered as a clinical decision support system and not an independent diagnostic system.

The objective of the study is as follows:

- The primary focus of this study is to propose a monitoring framework to assess their behavior using a deep learning model intelligently.
- In this study, we proposed a CNN-GRU model to classify into four classes of autism behavior: arm flapping, spinning, head banging, and normal Behavior.
- The effectiveness of the suggested approach has been assessed against various existing autism behavior monitoring approaches.

The remaining sections of the paper are arranged as follows. We will review the relevant work in Section 2. The CNN-based resources and techniques are explained in Section 3. Section 4 provides illustrations of the experimental findings, a comparative analysis, and discusses the paper's weaknesses and possible future study approaches. Section 5 presents the conclusion.

## 2 Related work

This section thoroughly summarizes the available and frequently used techniques to examine child Behavior and identify autism. Researchers have suggested several approaches to analyze and monitor Behavior to find autism. Some research used observation and video analysis to study motor Behavior.

### 2.1 Activity recognition based Behavior analysis

Activity recognition recognizes important occurrences in huge video collections. In order to identify clinically significant patterns from the photographs and videos and categories engaging activities for ASD youngsters, machine learning (ML) and computer vision (CV) have enhanced several features of human visual perception(Stevens et al., 2019; Zhao et al., 2022; Gu et al., 2018). Marinoiu et al. were presented with one of the largest multimodal autistic interaction datasets. Additionally, they suggested a fine-tuned action and emotion classification based on data gathered from children with ASD during robot-assisted therapy sessions. Their findings demonstrated a good agreement between machine-predicted scores and expert human diagnosis (Marinoiu et al., 2018). Rehg et al. presented a novel action recognition dataset to analyze children's social and communicative Behaviors using video and audio data. Their early experimental findings showed this dataset's potential to facilitate multi-modal activity detection (Rehg et al., 2013). Washington et al. suggested a deep learning-based computer vision classifier for identifying head banging in home recording videos. They use a head banging detector to extract the target head posture from videos, and then use a CNN + LSTM architecture to analyze the head banging motion. The experimental result enhanced deep learning models by attaining the accuracy of 90% in correctly identifying the head bagging and no head bagging (Rehg et al., 2013). They explored two methods and contrasted a bag-of-visual-words method with RNNs and CNNs. This demonstrates that deep learning architectures give outstanding results for detecting four activities: spinning, head banging, arm flapping, and other hand and arm movements (Washington et al., 2021). Ali et al. proposed a framework to recognize actions in videos of ASD children, using 3D Convolutional Neural Networks (3D-CNN), combined with target person identification and monitoring techniques. The experimental results show that deep learning models can attain an accuracy of 75% in correctly identifying autism Behavior actions in children (Negin et al., 2021). Rajagopalan et al. suggested the SSBD, which comprises videos of autistic kids carrying out everyday activities. They combined a histogram of optical flow alongside a histogram of dominating motions. Their proposed binary classification framework for headbanging and spinning has an accuracy of 86.6%, and for multi-classification, headbanging, arm flapping, and spinning have an accuracy of 76.3% (Rajagopalan and Goecke, 2014). Lakkapragada et al. proposed a deep learning-based model to classify autistic children abnormal hand movement and control hand movement in autistic children. This research aims to show that deep learning algorithms can successfully identify hand flapping in uncontrolled home videos. His work used the SSBD dataset and the deep learning model MobileNet. The experimental results show that the model has attained the highest accuracy of 84.0% (Lakkapragada et al., 2022). Tang et al. proposed a deep learning approach to detect emotion using smile facial expression. In this study, he presented the RCLA&NBH_Smile dataset, a novel dataset. Thirty-four baby face expression recordings of their interactions with their mothers were captured, and more than 77,000 frames were manually labeled. The experimental results show that the proposed model has attained the highest accuracy of 87.16% (Tang et al., 2018). Manocha et al. suggested a system for monitoring autistic children's physical activity to identify abnormalities. His study proposes an activity prediction algorithm constructed using a deep 3D CNN and LSTM for detecting physical anomalies. The experimental results show that the suggested approach has attained an accuracy of 92.89% (Manocha and Singh, 2019). Ali et al. created a Behavior diagnostic paradigm for ASD. The stereotyped Behavior of the children was recorded in an uncontrolled setting during their ASD diagnosis, and they gathered and interpreted a set of these recordings. Children with ASD will be classified and their performance assessed using a multi-modality-based late fusion network. The findings showed that the proposed approach achieves better results and an accuracy of 85.6–86.04% (Ali et al., 2022).

### 2.2 Facial expression-based behavioral analysis

People can communicate orally and nonverbally using facial expressions and eye contact. It can be distressing and lead to social anxiety for some individuals with ASD to maintain eye contact. It is difficult for kids with ASD to detect nonverbal clues, respond to them, and understand their gestures and emotions. Carpenter et al. used a trained computer vision model to extract positive, neutral, and various other facial features from a database of facial expressions. He argues that mimicking facial expressions is a crucial sign of social interaction abilities, supported by his discovery that kids with ASD exhibit more neutral facial expressions (Carpenter et al., 2021). He proposed a transfer learning-based approach to analyzing the facial features of autism patients. Their research showed that their method could more accurately identify the emotional expressions of children with ASD (Han et al., 2018). He developed an automated approach to identify emotions in youngsters engaging with robots to treat ASD. According to their research, computer vision could improve Behavior analysis when people engage with robots (Leo et al., 2015). Leo et al. suggested a machine learning approach for examining the facial expressions of kids with TD and ASD. The suggested approach may be effectively used to analyse the facial expressions made by kids with ASD thoroughly. Their results showed that their approach may yield an F1-score of 0.86% (Leo et al., 2019). Chintan et al. suggested an approach for digitally interpreting facial expressions to analyze human Behavior. He predicted the emotion across seven distinct categories using a deep learning network and the CK+ dataset (Thacker and Makwana, 2019). Ahmed et al. developed an automated approach to identify kids' facial expressions in videos recorded while they were using computer-assisted learning software. Their results showed how computer vision evaluation can automatically quantify Behavioral and emotional participation (Ahmed and Goodwin, 2017). Furthermore, a deep learning technique for facial image analysis has been used to identify cognitive developmental issues. Li et al. introduced a CNN-based approach for classifying ASD based on face features. Its results show that several face features improve autism classification (Li et al., 2019). Every methodology covered in the literature review section has been employed in various deep learning or machine learning models, except for a few limitations that are described in the comparison in Table 1. Most research tested their suggested model utilizing performance assessment parameters; there was room for improvement, evident in our results section.

**Table 1:** Summary of related work.

**References**	**Dataset**	**Method**	**Accuracy**	**Limitation**
Stevens et al. (2019)	AVA benchmark, JHMDB, UCF101	CNN	75.3%, 92.8%, 84.8%	He proposed a human action dataset to compare the performance with two different datasets, but did not describe the pre-processing and model development steps.
Zhao et al. (2022)	DE-ENIGMA	CNN	45.68%	Need to improve results
Gu et al. (2018)	Multimodal dataset MMDB	SVM	Not reported	Need to improve methodology.
Marinoiu et al. (2018)	SSBD	CNN + LSTM	90.77%	He implemented a basic CNN model that performs binary classification and was tested on a small dataset.
Rehg et al. (2013)	ESBD	SVM, MLP, LSTM, NB	79.28%	Need to improve results.
Washington et al. (2021)	SSBD	3D CNN	75.62%	Need to improve results.
Carpenter et al. (2021)	Own dataset	Statistically analysis	73.0%	Need to improve methodology. No information on data pre-processing. Need to improve results.
Ali et al. (2022)	FER	Transfer learning	82.2%, 87.1%	He proposed a framework to analyze emotion-based Behavior, but did not describe the pre-processing and model development steps.
Leo et al. (2015)	FER	CNN + SVR	86.0%	Not an evaluation of real-time expression-based Behavior analysis.
Rajagopalan and Goecke (2014)	SSBD	LSTM, MobileNetV2	85.0%	He implemented pretrained models that perform binary classification and tested them on a small dataset.
Lakkapragada et al. (2022)	RCLA&NBH Smile data	CNN	87.16%	He implemented a basic CNN model that performs binary classification and was tested on a small dataset. Other autism Behavior landmarks are ignored. Need to improve methodology and do multi-classification.
Tang et al. (2018)	Own dataset	3D CNN-LSTM	92.89%	Autism associated Behavior landmarks and features are not used in the dataset.
Howard et al. (2017)	SSBD	VGG 19, LSTM	96%	He implemented pretrained models that perform binary classification. Need to improve methodology and do multi-classification.

## 3 Methodology

**Figure 1:**
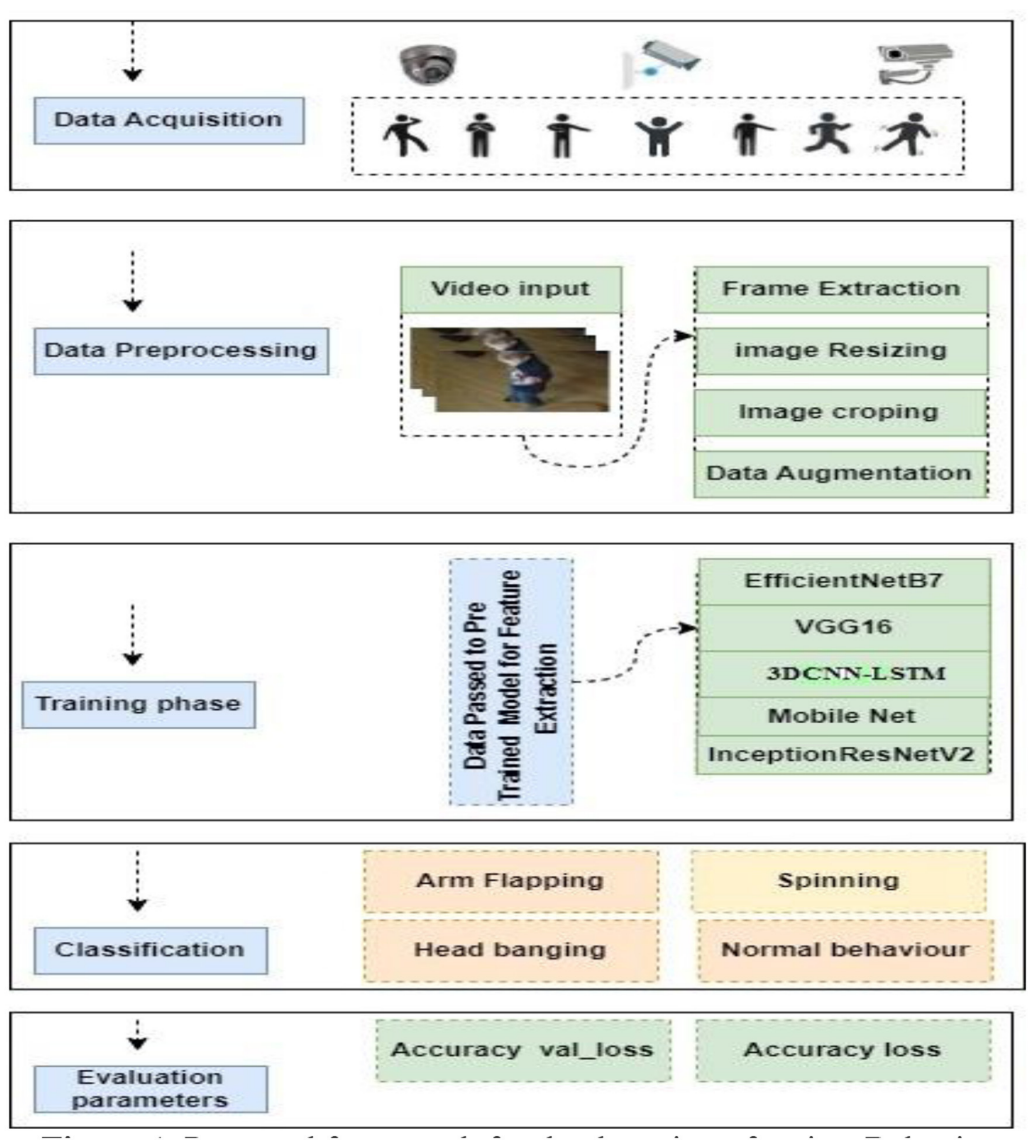
Proposed framework for the detection of autism behavior.

In this section, we describe our proposed approach in detail. We used several deep learning techniques to monitor Behavior and classify the autism related symptoms and normal Behavior. The primary stages of our suggested approach are shown in Figure 1; further information is given below.

### 3.1 Data acquisition

**Figure 2:**
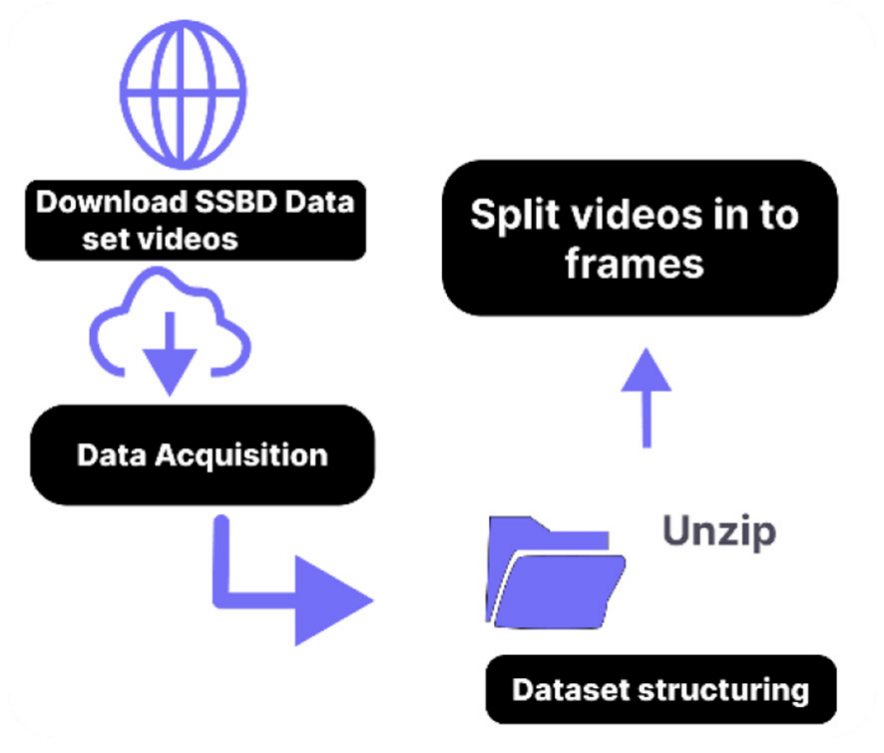
Flow chart for dataset preparation process.

**Figure 3:**
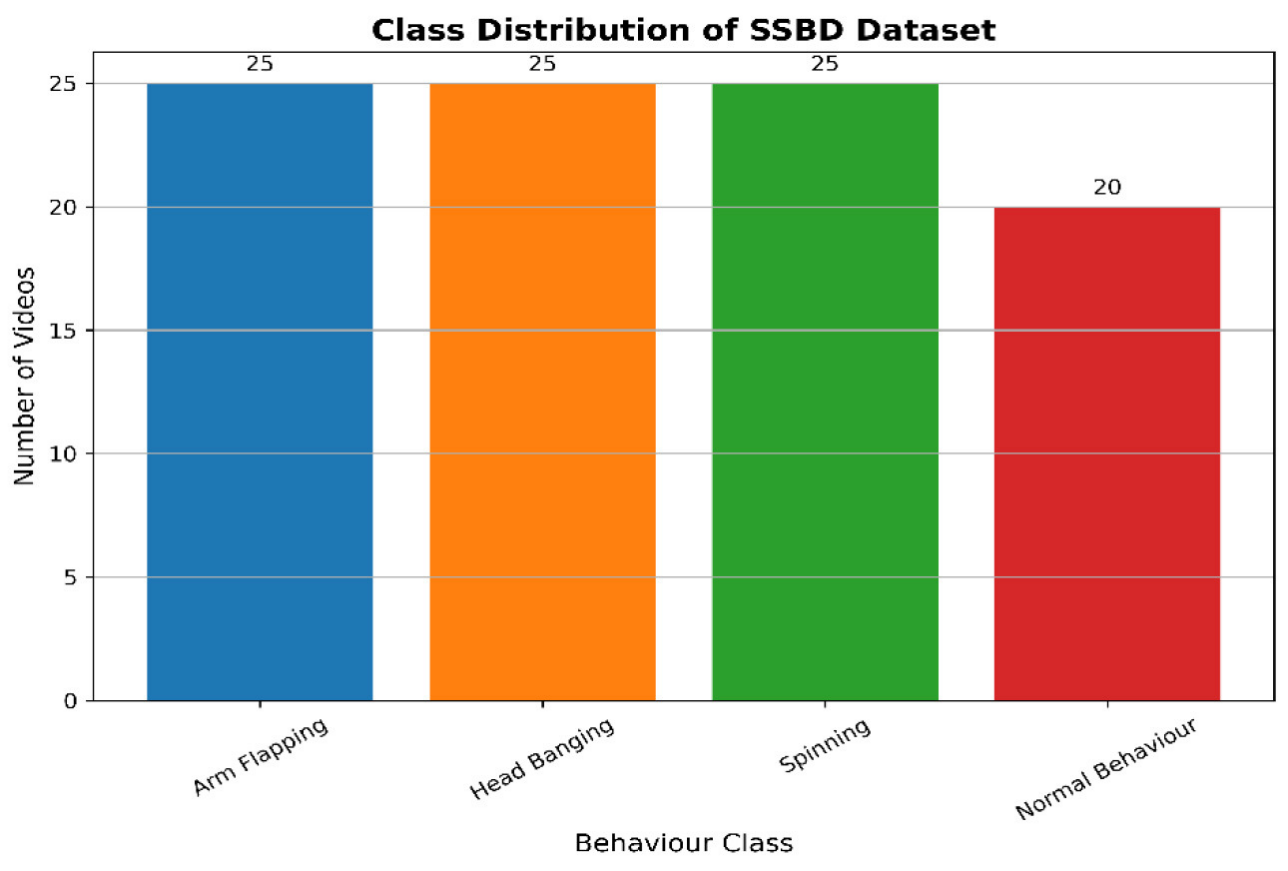
Class distribution of dataset.

In data acquisition, we collected the dataset used in our experiment. There is no previous dataset available that fully meets our condition. The SSBD data set was used to train the deep learning models. The SSBD is a freely accessible dataset gathered from autistic children. Figure 2 presents the detail steps for data acquisition and depict the flow of converting video into frames. The data consists of Videos that capture autistic children performing actions like spinning, headbanging, and shaking their hands. Parents and caretakers posted their videos on websites that are accessible to the public. Figure 3 presents the details of classes in dataset.

There are 75 videos in the preliminary dataset, which was gathered from the social media platform YouTube (Rajagopalan et al., 2013). Only one dataset, SSBD, is freely available, but this data is somewhat helpful. However, it does not fully meet our conditions. The SSBD dataset was developed by collecting the naturalistic video recordings of autistic children.

### 3.2 Data pre-processing

#### 3.2.1 Frame extraction

**Figure 4:**
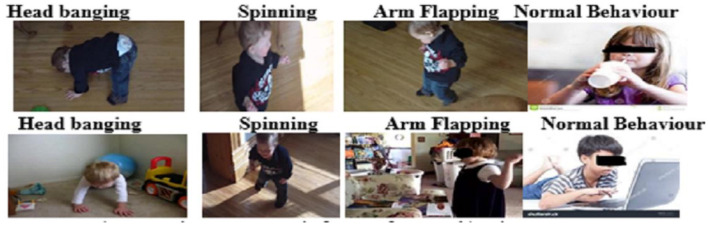
Some sample frames of the SSBD video dataset.

The developers of the SSBD dataset gave the URLs of 75 YouTube videos, with annotations at the start and finish of abnormal Behavior that suggests autism. Each video has an average length of 90 s. There are multiple Behavioral movements in various time throughout the videos in this dataset. As a result, it was necessary to divide the portions according to the Behavioral movements, such as autistic Behavior, head banging, spinning, arm flapping, or other normal Behavioral movements. Every video was just a series of repeated frames meant to provide the impression of motion. The Python OpenCV package was use from each video to extract the frames and pre-process them. All videos were uniformly sample to obtain 30 frames per video as to maintain a temporal variation across all the samples. The frames were extracted and convert into feature vectors and a temporal sequence is form by placing the feature vectors in consecutive order. This sequence had a constant input length of GRU of 30 time steps, is use as input to capture temporal dependencies for classification. Figure 4 displays some frames of videos from the SSBD dataset.

#### 3.2.2 Image resizing and cropping

All the sizes of the images in each class are different, so they are not all the same. Therefore, all photos were transformed to 224 × 224-pixel values to achieve linearity and better outcomes. Another issue with images is that several unnecessary objects surround the object of interest. This problem results in an inaccurate categorization of the actual Behavior class. So, we solve the issue by extracting the region of interest and drawing a bounding box around the desired object using the OpenCV package. We use the bounding box detection to crop the original images.

#### 3.2.3 Data augmentation

Data augmentation was used on the data samples in order to produce more image samples. Applying this strategy has the primary benefit of increasing model prediction and accuracy. We used the image data generator function from the Python Keras library to reduce over-fitting and increase the diversity of the training set. Reducing the variance in pixel values would enhance the computer's performance. Pixel values can only be found in the range [0, 1] by default due to the input value 1/255. The images were rotated toward a 25-degree targeted orientation. An unrestricted right and left image rotation is possible with the width shift range transformation when the width shift value is set to 0.1. Additional data sets were shifted with a zoom range of 0.1 in vertical and horizontal directions Every frame of the videos was augmented with rescaling, random rotation (maximum 20), width and height perturbation (maximum 20 percent), shear (maximum 20 percent), zoom (80–120 percent), as well as random horizontal flipping and nearest-pixel filling performed after perturbation. These additions brought in further data variety and were used to enhance model generalization (Howard et al., 2017).

## 4 Deep learning models

This research presents the practical use of deep learning models for video-based human Behavior recognition and classification. On the other hand, videos consist of a series of images placed in a particular order to create an activity. Various techniques, including long short-term memory networks (LSTMs), GRU, MobileNet, VGG19, and Efficient NetB4, could be applied to the classification of videos (Khalil et al., 2022). The primary goal was to classify autistic Behavior with satisfactory results. This is why I have experimented with several deep learning models, applying the pre-processing steps listed above on data sets before giving input to any model.

### 4.1 MobileNet

The MobileNet model is a lightweight model designed explicitly for embedded and mobile devices with constrained computational capabilities, designed by Andrew G in 2017. The MobileNetV2 paradigm was chosen based on several different considerations. Due to the small dataset used to train the model, over-fitting was possible. However, utilizing a smaller but more expressive system, such as MobileNetV2, greatly reduced this effect. The primary concept included employing depth-wise distinct convolutions to minimize processing demands and parameter values while achieving high precision (Howard et al., 2017).

### 4.2 VGG19

University of Oxford researchers introduced the VGG (Visual Geometry Group) network as their deep convolutional neural network model in 2014. The VGG network model features 19 distinct layers, where 16 belong to convolutional operations, followed by fully connected layers as the final three. The stride measures at one pixel, and the pad measures at one pixel with a filter size set to 3 × 3. A small kernel size enables parameters to cover the entire image space while reducing the number of parameters. The 2 × 2 max pooling function of VGG-19 executes through a stride of 2. VGG Net further supported the theory that convolutional neural networks need an extensive layer structure because they understand visual patterns through structured systems. Feature extraction takes place through 16 layers of the network, following classification functions operated by the first three convolutional layers. Each feature extraction layer group contains five sections where max pooling operates as the final step. The system requires images with 224 × 224 dimensions (Simonyan and Zisserman, 2015).

### 4.3 Efficient NetB4

Tan and Le proposed the EfficientNet-B4 as a convolutional neural network (CNN) model in 2019, which became part of the EfficientNet family. The image classification domain has recognized the significant accomplishments of EfficientNet-B4. The platform contains two scaling elements for reliable dimension expansion and resolution enhancement, resulting in cutting-edge performance. The neural network contains depth-wise separable inverted bottleneck convolutions named MBConv and squeeze-and-excitation SE blocks for feature recalibration improvement (Szegedy et al., 2015).

### 4.4 LSTM

An LSTM model (also known as a Long Short-Term Memory) is a type of recurrent neural network that is specifically trained to acquire long-term dependencies in sequential data. It solves the vanishing gradient issue on the basis of memory cells and gating. Time-series analysis, and natural language processing are the most effective tasks with which LSTMs work. The model has input, forget and output gates that selectively remember or forget information. This is an advantage because LSTM models are highly appropriate in modeling intricate patterns of time-varying data (Saar-Tsechansky and Provost, 2007).

### 4.5 3D CNN

The 3D CNN is a continuation of the traditional 2D CNN, which operates on a three-dimensional dataset. It takes into account both time and space using 3D convolutional kernels. The applications of 3D CNNs include video analysis, action recognition and medical imaging. They model dynamic behaviors by learning motion patterns across sequential frames. This is why 3D CNNs are applicable in those tasks that require spatiotemporal features mentions (Ali et al., 2023).

### 4.6 Gated recurrent unit

**Figure 5:**
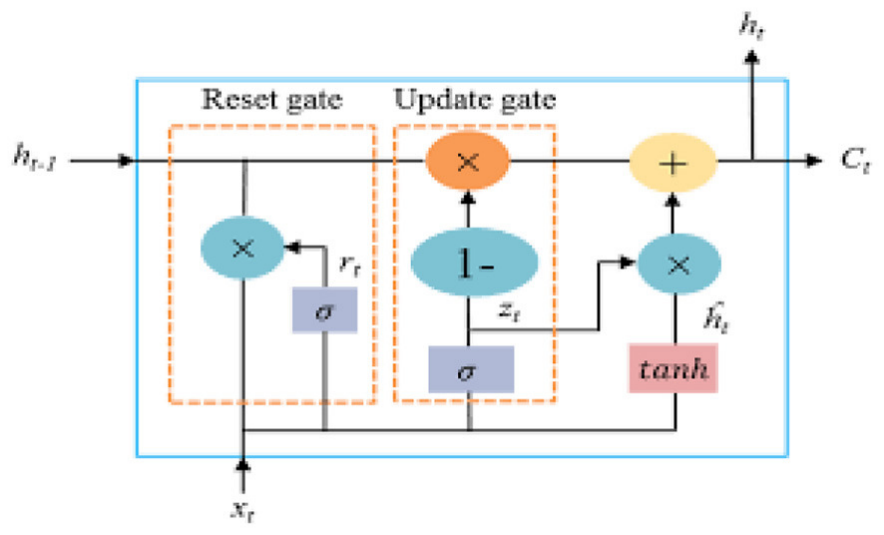
Structure of GRU model.

The GRU model, a particular kind of recurrent neural network (RNN), is a widely used deep learning technique. GRU was developed to address the vanishing gradient issue with RNN. GRU controls the transmission status according to the state of the gates to remember the information that needs to be stored for an extended period, and less important information. Figure 5 illustrates a GRU cell's basic structure, consisting of two gates: an update gate and a reset gate. Information moving through the cell is rejected and accepted by the two gates. The reset gate determines what quantity of the previous data should be forgotten based on the decision taken by the sigmoid function σ. The output of the sigmoid is *r*_*t*_. The GRU model will process the data if the sigmoid function value is 1; if it is 0, the data will not be processed. The current input *x*_*t*_ and the prior hidden state (ht-1) serves as the input for the reset gate. The Update gate determines the information that is going to be modified to convey a future state. Thus, the update gate requires a fundamental aspect of the prior state. To modify the cell state, the update gate additionally incorporates a sigmoid activation function (Zhang et al., 2015; Manocha and Singh, 2019; Bansal et al., 2023).

### 4.7 Proposed CNN GRU model

**Figure 6:**
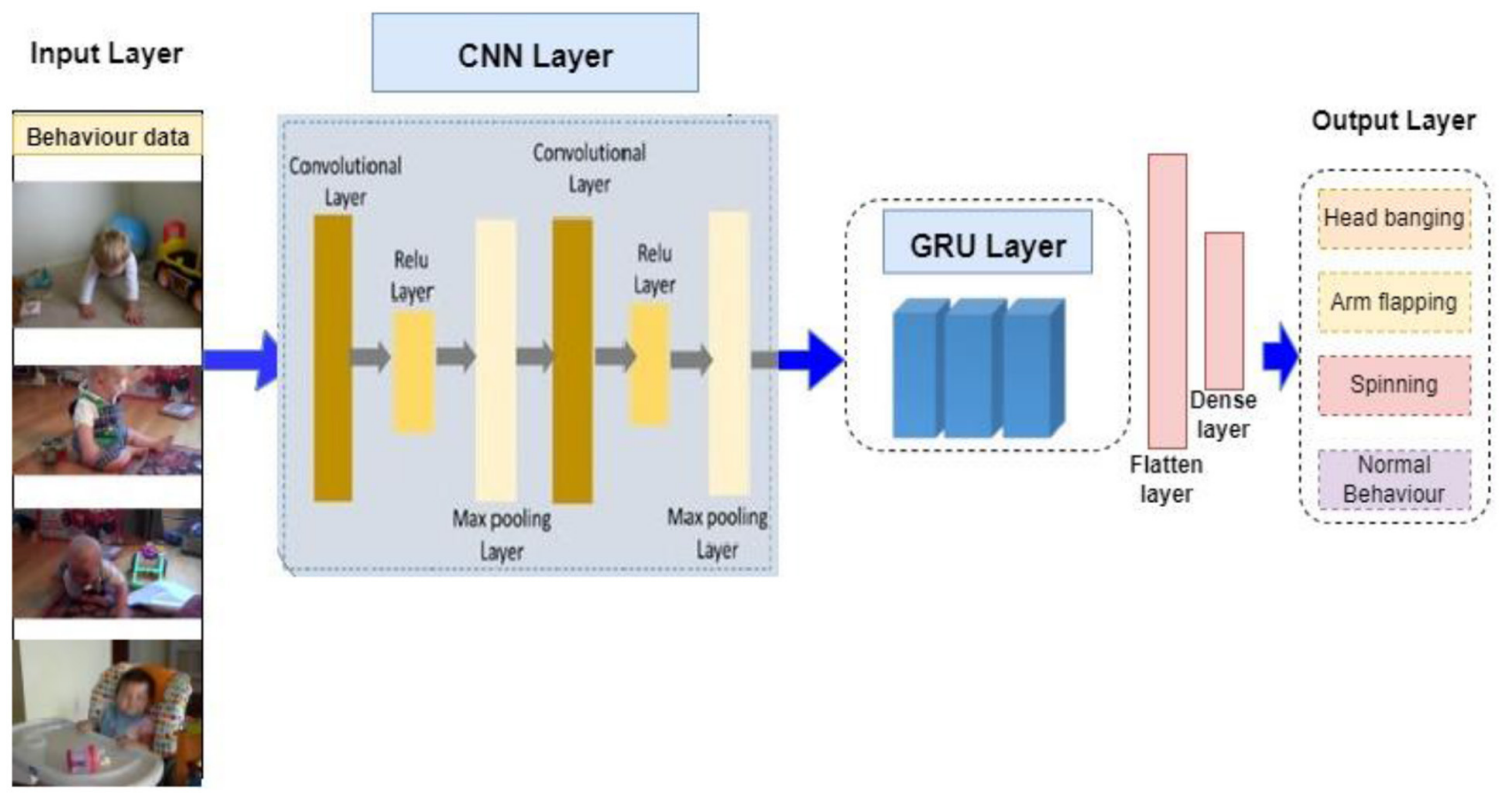
Proposed architecture of the CNN-GRU model.

In this investigation of autism action classification, CNN in conjunction with GRU was employed as a classifier. Compared to the LSTM architecture, the GRU architecture is simpler and requires fewer parameters to be configured. In Figure 6, we show the proposed CNN GRU model architecture. The CNN GRU model uses a CNN convolutional layer to extract some key features from an image while preserving the image's original feature layout. Additionally, to avoid the issue of overfitting the model, the max pooling layer is utilized to shave the weak feature values and choose the deeper feature values from the key instances. This work also employed the rectified linear unit to trim down the eigenvalues smaller than 0 between the convolution layer and the layer that performs max pooling to speed up model training. The gated recurrent unit's (GRU) update and reset gates are then used to process the eigenvalues, speeding up the model's computation and improving its accuracy. For the convenience of the fully linked layer's use later on, the feature value is converted into a single-dimensional data by connecting the flattened layer. Lastly, the output is generated using the Softmax activation function.

The k-fold validation results and the comparative performance analysis, which is strongly support the selection of CNN-GRU architecture. Although layers in CNN are useful in extracting spatial features of single frames, autism-related Behavior is a temporal phenomenon that need sequence modeling across frames. GRU was chosen in place of LSTM because it uses a simpler gating operation and fewer parameters to be trained, resulting in better training stability and less overfitting during a k-fold training where the same model is trained repeatedly on varying data splits. Based on the results of the performance tables, CNN-GRU model is always more precise, recall, and F1-score per-fold than standalone CNN models, which means that it generalizes better and its performance is less variable. This equal performance at least in recall and F1-score indicates that GRU is very good at capturing the temporal dependencies, but with no computational cost of LSTM. Thus, the k-fold validation findings are empirical reasons to use CNN-GRU as an effective and strong architecture to assess autism behavior.

### 4.8 Hyperparameters optimization

The same hyperparameter settings were used to train all the models to facilitate a fair comparison of them: VGG16, CNN-LSTM, MobileNet, CNN-GRU and EfficientNet-B7. The output layer activation function was the Softmax and a dropout rate of 0.4 was used to ensure that it did not overfit. The Adam optimizer was used with the learning rate of 0.001 to train the models to minimize the categorical cross-entropy loss. The evaluation metric was the accuracy and all models were trained with 50 epochs and under the same conditions.

### 4.9 Performance evaluation measure

Performance evaluation is necessary in finding out the extent to which a classification model achieves its objectives. The target performance evaluation metrics determine how well and accurately the model works on the test data. An appropriate selection of metrics, such as Accuracy, and F1 score, among others, is essential to carry out a detailed assessment (Zhang et al., 2015). The following are the formulae generally used to calculate the above performance measures.

Accuracy is calculated as the sum of the value of TP and TN, divided by the total number of TP, TN, FP, and FN values.


$$\begin{array}{l} Accuracy=\frac{\text{T}\text{P}\;+\text{T}\text{N}}{\text{T}\text{P}\;+\text{T}\text{N}+\text{F}\text{P}+\text{F}\text{N}} \end{array}$$ (1)

The percentage of correctly determines the degree of precision recognized positive instances and dividing the total number of positively and mistakenly identified positive instances, as formulated in Equation 2.


$$\begin{array}{l} Precision=\frac{\text{T}\text{P}\;}{\text{T}\text{P}\;+\text{F}\text{P}} \end{array}$$ (2)

Recall is calculated as the percentage of correctly recognized positive instances, divided by the total number of true positive and identified false positive instances, as formulated in Equation 3.


$$\begin{array}{l} Recall=\frac{\text{T}\text{P}\;}{\text{T}\text{P}\;+\text{F}\text{N}} \end{array}$$ (3)

F1 score is calculated by multiplying the mean of Recall and Precision and dividing by the total number of Recall and Precision, as expressed in Equation 4.


$$\begin{array}{l} F1-score=\frac{2\text{T}\text{P}\;}{\text{F}\text{N}\;+\;\text{F}\text{P}\;+\;2\text{T}\text{P}} \end{array}$$ (4)

## 5 Results and discussion

The experiment is conducted via Google Collaboratory, an open-source cloud-based tool provided by Google. This section presents the results of our suggested CNN-GRU model and other deep learning models on a publicly available autism Behavior dataset. A variety of deep learning models were tested for validated accuracy, and based on the findings, more refinement was made to the selected model customization based on validation accuracy. Moreover, we assess a customized suggested CNN-GRU classifier using performance metrics such as accuracy, Specificity, Sensitivity, precision, recall, and F1-score.

### 5.1 Performance analysis of proposed model

In Table 2 presents the results of different deep learning models. We implemented five deep learning models on the SSBD dataset and assessed their performance using validation accuracy and loss. Our proposed customized CNN-GRU model performs better than other models and attains the highest validation accuracy of 96% with a minimum validation loss of 0.16. Moreover, VGG16 performs the worst compared to others and achieves a minimum validation accuracy of 72.37% with a validation loss of 4.75. Our suggested approach has attained the highest accuracy score of 0.96 for arm flapping and head banging classes. In contrast, normal Behavior and spinning classes have attained low performance metrics scores of 0.96 and 0.97.

**Table 2:** Performance evaluation of different deep learning models on the SSBD dataset.

**Dataset**	**EM**	**VGG16**	**MobileNet**	**Efficient Net B7**	**3D-CNN + LSTM**	**CNN-GRU**
SSBD	Val accuracy	0.89	0.95	0.95	0.89	0.96
	Val loss	0.42	0.39	0.38	0.40	0.16

**Figure 7:**
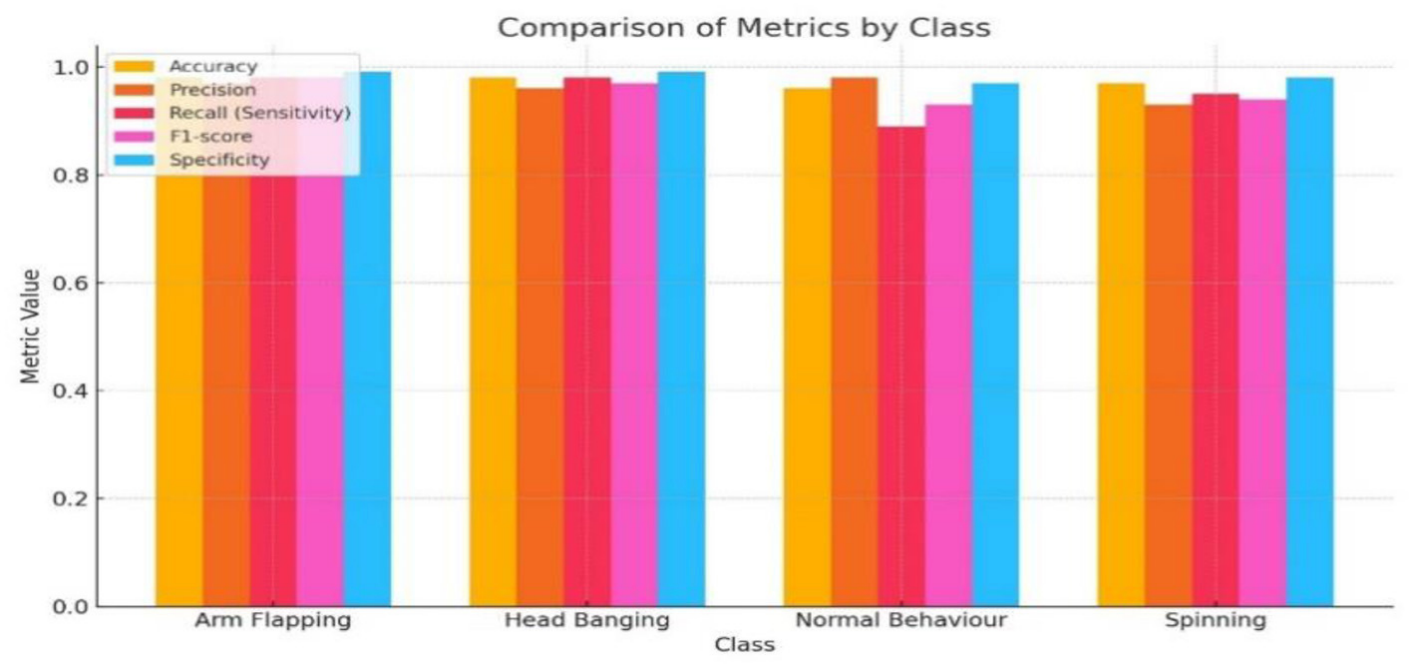
Class wise performance assessment of proposed CNN-GRU model.

The graphical representation of the experiment results of our proposed CNN-GRU model is shown in Figure 7. We clearly see that the Arm flapping class has attained the highest bar because it attains the highest F1 score, 0.98, and the head banging class achieves the same result. Normal Behavior and spinning classes achieve the lowest bar because they earn low F1 scores of 0.93 and 0.94, respectively.

#### 5.1.1 Confusion matrices analysis

**Figure 8:**
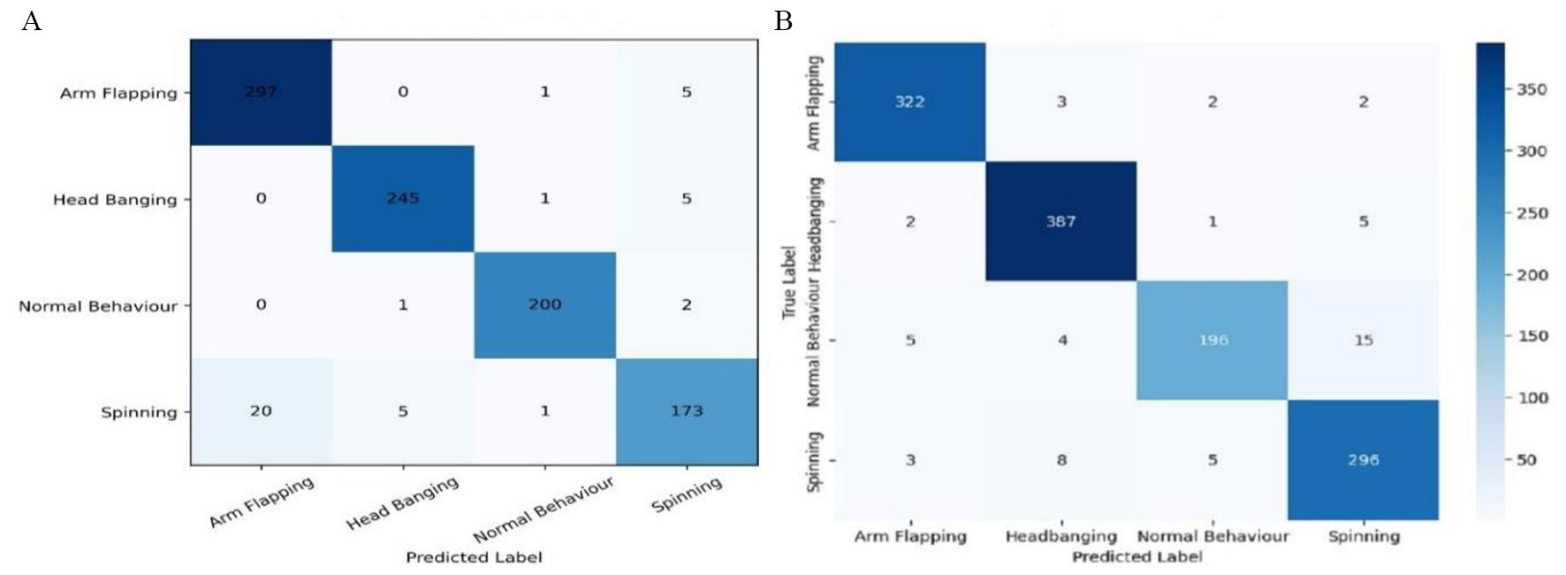
Confusion matrices of proposed CNN GRU model **(a)** k-fold cross validation results **(b)** over all test performance.

In Figure 8 show the confusion matrices of the four behavior classes of autism Arm Flapping, Head Banging, Normal behavior, and Spinning. In Figure 8a result of k fold validation of CNN-GRU shows that there is a strong and balanced performance in the classification of all four classes of behaviors. There is a very high level of correct prediction with 297 out of 303 samples being correctly predicted and a slight level of confusion with Spinning and Normal Behavior which implies that there is strong feature learning of Arm Flapping. Head Banging also demonstrates good performance with 245 correct classifications with very few misclassifications with a majority of them as Spinning and therefore there is a little overlap in motion patterns but good separability. Normal Behavior is very stable with the least number of misclassifications. Spinning has the highest false negative rate (~14%), which means that it is the most difficult to learn. In Figure 8b CNN GRU test results, Arm Flapping (322 correct, 7 incorrect) and Head Banging (387 correct, 8 incorrect) had a high class wise accuracy (0.98). Normal Behavior 196 correct predictions, 24 misclassifications (0.96 accuracy). Spinning has made 69 correct predictions and 16 misclassifications suggesting a high overall recognition and a relatively high level of confusion. The normalized confusion matrix, the majority of misclassifications are between Spinning and the repetitive behaviors (Arm Flapping and Head Banging), so there is a possible overlap in the dynamics of motion. Normal Behavior is moderately confused with repetitive classes in the independent test set. Table 3 presents the performance assessment analysis of several deep learning models regarding recall, F1-score, and precision. Our proposed CNN-GRU model performs better than other models and attains the highest precision score of 96%, a recall of 96%, and an F1 score of 96.31%. Conversely, the VGG16 model had a minimum precision score of 77.04%, a recall of 52.74%, and an F1 score of 51.95.

**Table 3:** Performance comparison of the proposed customized CNN-GRU model with baseline classifiers.

**EM**	**VGG16**	**MobileNet**	**Efficient Net B7**	**3D-CNN + LSTM**	**CNN-GRU**
Precision	0.91	0.93	0.95	0.89	0.96
Recall	0.90	0.92	0.95	0.89	0.95
F1-score	0.90	0.92	0.95	0.89	0.96

**Figure 9:**
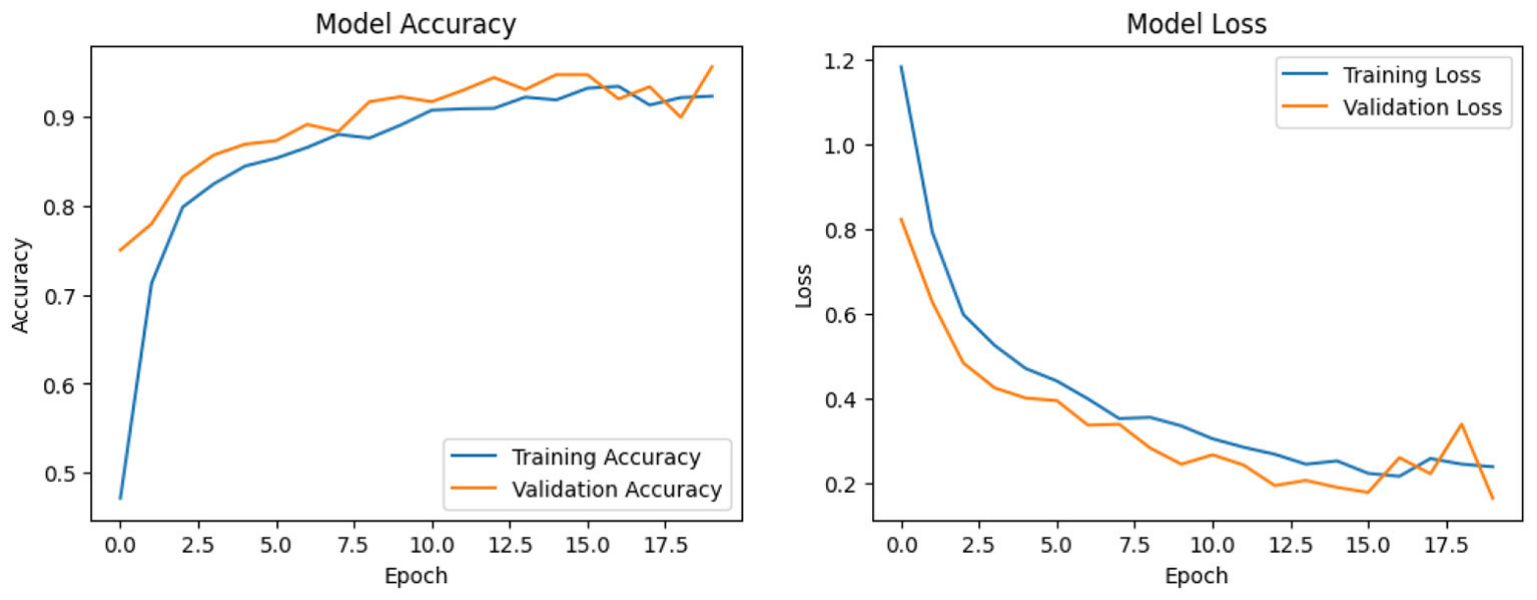
Learning curve of proposed CNN GRU model.

In Figure 9, we depict the model learning curve of our suggested approach. The above figure is divided into two separate images. The learning curve for the loss of training and validation is displayed on the left portion of the image. The image's right portion shows the training and validation accuracy learning curve. These learning curves are highly helpful since they provide insight into how a model behaves in terms of miss rate and learning ability.

**Figure 10:**
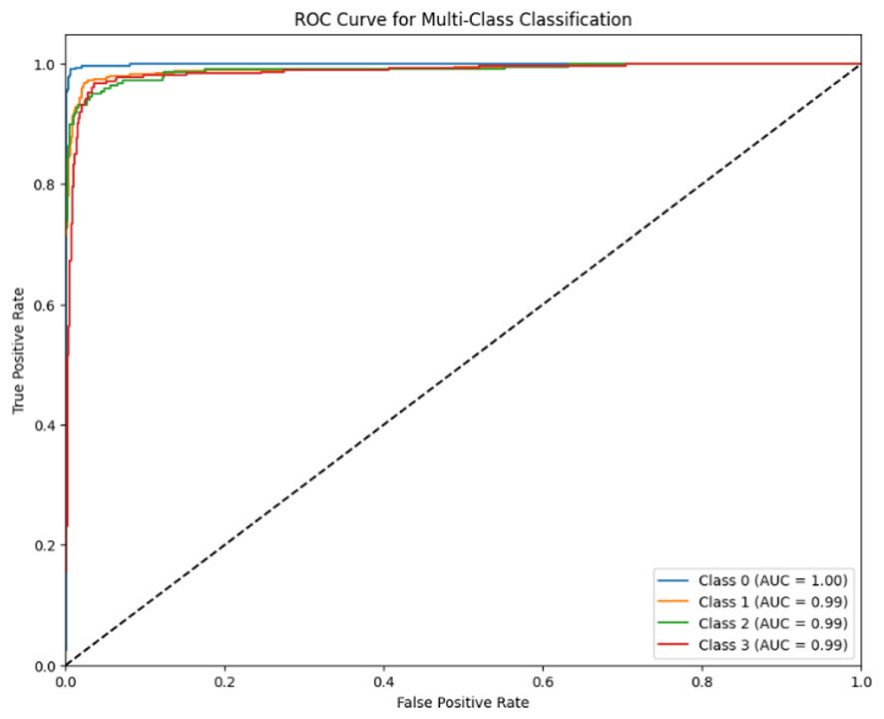
ROC curve of proposed CNN GRU model.

The ROC curve of the Proposed CNN GRU Model is displayed in Figure 10. This curve graph's primary benefit is its ability to visualize model performance, highlighting the trade-off between the rate of false positives and true positives. Because our suggested model was multiclassified, the ROC curve displays four distinct behavior classes.

**Figure 11:**
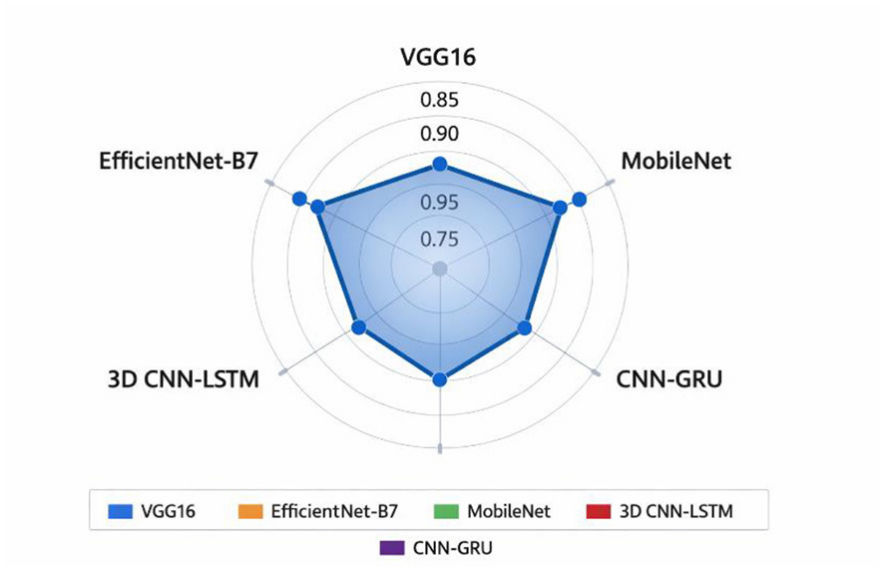
Accuracy comparison of the proposed CNN GRU model with baseline classifiers.

The Figure 11 shows the relative efficiency of various classifiers, which demonstrates the usefulness of the suggested CNN-GRU model. However, CNN-GRU was the most accurate of all the five analyzed models with the highest value of 0.9294 and the least variance (standard error of 0.0038), which implies the best performance and the least variation. MobileNet (0.9193 ± 0.0078) and VGG16 (0.9142 ± 0.0131) were also found to be very strong but a little less than CNN-GRU. Conversely, EfficientNet-B7 (0.8808 ± 0.0084) and 3D CNN-LSTM (0.8912 ± 0.0105) had relatively lower accuracies implying that they were not very effective in this task. It is important to note that 3D CNN-LSTM is video-based model but CNN-GRU was more accurate than CNN-LSTM showing the benefit of combining CNN features extractions with GRU to model time. On the whole, the radar chart visually highlights that CNN-GRU model has a high level of accuracy and consistent performance; thus, it is the strongest among the analyzed architectures.

### 5.2 2 K fold cross validation analysis

All the models were tested by means of k-fold cross-validation to ensure a high quality and objective evaluation. In Table 4 presents the results of different deep learning models. The findings suggest that the CNN-GRU model has the best overall performance with the average accuracy of 0.92 and equal precision, recall and F1-score of 0.91. MobileNet is also highly and steadily performing, with 0.91 on all evaluation measures, which demonstrates its efficiency and the ability to generalize in conditions of cross-validation. VGG16, is also highly and steadily performing, with achieving score 0.91. EfficientNet-B7 achieves the competitive performance of 0.88 and the standalone 3dCNN-LSTM model achieves the same of 0.89. Comprehensively, the stability and the generalization capabilities of the hybrid CNN-GRU model are supported by k-fold cross-validation and determine it as the most trustworthy strategy among the considered ones.

**Table 4:** K fold comparison of the proposed CNN GRU model with base line models.

**Classifiers**	**Accuracy**	**Precision**	**F1-score**	**Sensitivity**	**Specificity**
Vgg16	0.9142 ± 0.0131	0.9146 ± 0.0123	0.9138 ± 0.0125	0.9142 ± 0.0131	0.9146 ± 0.0123
Efficient Net b7	0.8808 ± 0.0084	0.8799 ± 0.0092	0.8786 ± 0.0093	0.8808 ± 0.0084	0.8799 ± 0.0092
Mobile Net	0.9193 ± 0.0078	0.9191 ± 0.0080	0.9189 ± 0.0078	0.9193 ± 0.0078	0.9191 ± 0.0080
3dCNN-LSTM	0.8912 ± 0.0105	0.8927 ± 0.0083	0.8888 ± 0.0123	0.8912 ± 0.0105	0.8927 ± 0.0083
CNN-GRU	0.9294 ± 0.0038	0.9290 ± 0.0036	0.9284 ± 0.0039	0.9294 ± 0.0038	0.9290 ± 0.0036

### 5.3 Statistically analysis

The significance level (alpha) is set to 0.05. The proposed CNN-GRU model is compare to baseline models by using four paired *t*-tests. All the comparisons had *p-*values that were significantly lower than the adjusted significance value of 0.0125 as indicated in Table 5, which proved to be statistically significant. Precisely, CNN-GRU had tremendous improvements compared to MobileNet (*p* = 0.0028) and VGG16 (*p* = 0.0051). Greater significance is found as compared to 3D CNN-LSTM and EfficientNet-B7, and lower p values. These findings suggest that the high performance of CNN-GRU model is not random and is not due to chance alone. Bonferroni's correction is use to regulate the family wise error rate. The significance value is adjusted using Equation 5.


$$\begin{array}{l} \alpha_{\text{adjusted}\;}=\;\frac{\alpha }{\text{N}} \end{array}$$ (5)

**Table 5:** Statistically analysis of proposed CNN GRU model with base line models.

**Classifiers**	***T-*test**	***P-*test**	**Significance < 0.0125**
CNN-GRU vs. MobileNet	3.6811	0.0028	Yes
CNN-GRU vs. VGG16	3.5239	0.0051	yes
CNN-GRU vs. 3D CNN-LSTM	10.8180	0.0000	yes
CNN-GRU vs. EfficientNet-B7	16.6697	0.0000	yes

### 5.4 Ablation of study

In this ablation study, the value of each specific architecture element is analyzed by comparing the proposed CNN-GRU model with a variety of baseline and hybrid networks, i.e. VGG16, EfficientNet-B7, MobileNet, 3D CNN-LSTM. We measures the importance of various architectural elements through a comparison of the proposed CNN-GRU model and various baseline and hybrid networks such as VGG16, EfficientNet-B7, MobileNet, and 3D CNN-LSTM. Table 4 summarizes quantitative results in terms of accuracy, precision, recall and F1-score whilst Table 5 displays statistical significance of performance differences using paired *t*-tests. As Table 4 indicates, the proposed CNN-GRU model is the most overall winner in terms of the accuracy of 0.9294 ± 0.0038 and F1-score of 0.9284 ± 0.0039, surpassing all other competing models. Also, the t-test results in Table 5 statistically prove the improvements, with CNN-GRU showing significant performance improvements over MobileNet (*t* = 3.6811, *p* = 0.0028) and VGG16 (*t* = 3.5239, *p* = 0.0051). Moreover, comparatively with spatiotemporal architectures, CNN-GRU demonstrates a substantial and statistically significant edge over the 3D CNN-LSTM (*t* = 10.8180, *p* < 0.001), which means that GRU offers to represent time more efficiently and more reliably than LSTM in such a scenario. The most significant statistical support is seen against EfficientNet-B7 (*t* = 16.6697, *p* < 0.001) indicating that the lack of explicit temporal modeling is not offset by the increase in depth of the network.

### 5.5 Comparative analysis with existing method

It is a challenging endeavor due to autism diagnosis relies on long-term observation of human behavior. In this study, we presented an intelligent approach to detect autism related Behavior such as hand flapping, head banging, spinning, and normal Behavior. Our proposed approach uses deep learning and computer vision techniques. An analysis of comparisons is necessary to demonstrate the effectiveness of the suggested model. In Table 6, we present the details of the comparative analysis of our suggested models with various current models used in recent studies. After examining and comparing the performance of our suggested model with various current models, we found that the proposed model achieves a 92.94% accuracy score, which is far better than other approaches used in recent studies.

**Table 6:** Comparison of the proposed model with relevant studies.

**References**	**Dataset**	**Method**	**Accuracy**
Washington et al. (2021)	SSBD	3D CNN	75.62%
Marinoiu et al. (2018)	SSBD	CNN + LSTM	90.77%
Stevens et al. (2019)	SSBD UCF101	KNN	86.6% 76.3%
Rajagopalan and Goecke (2014)	SSBD	LSTM, MobileNetV2	85.0%
Lakkapragada et al. (2022)	RCLA&NBH	CNN	87.16%
Wei et al. (2023)	SSBD	CNN	83%
Park et al. (2024)	SSBD	LRCN	79.61%
Prakash et al. (2025)	SSBD	DCNN	78.57%
Proposed	SSBD	CNN-GRU	92.94%

## 6 Discussion

The comparative analysis reveals that the suggested CNN-GRU model is more successful than base architectures in recognition accuracy and resilience. Its high accuracy, recall, and F1-score suggest that it has a balanced performance in terms of correctly identifying behavioral classes with the lowest error predictions. The traditional CNN models (e.g., VGG-based models) demonstrate a relatively lower recall and F1-scores, implying that they are unable to capture complex behavioral patterns. Though lightweight and deep learning models like MobileNet and Efficient Net are also competitive, they still do not outperform the proposed model. The consistency of the CNN-GRU architecture in terms of its accuracy and F1-scores across folds are also supported by the K-fold cross-validation results. Conversely, the stand alone 3D CNN-LSTM model shows relatively low performance that temporal modeling is not sufficient without strong spatial feature extractions. Moreover, the lower variance between folds shows that the model is less sensitive to changes in data distortions, which is especially critical in real-world situations of studying behaviors.

### 6.1 Real-world clinical setting

Instead of being a stand-alone diagnostic system, the suggested system shows tremendous potential for real-world clinical implementation as a decision-support tool. In practice the proposed CNN-GRU-based system to be use as a decision support system for clinicians, integrated with the existing screening system. In the regular Behavioral screening of patients, video clips of the children recorded with the use of cameras in clinical setting and interpreted by the system to provide risk scores and behaviors for Autism. These risk scores and Behaviors can easily integrated into existing electronic health records (EHRs) software systems/clinical dashboards for the clinician to interpret the predictions of the system alongside existing screening tools. In terms of system implementation, the system would not be hardware-dependent; it would be able to run on regular clinical computer workstations or be deliver through a cloud-based system for easy accessibility. The system also would be capable of near-real-time predictions due to the computational efficiency of the CNN-GRU architecture, making it ideal for use in early screening settings. The system would be intend to improve the screening process, help with regular screening, and facilitate the clinician with the diagnosis of ASD in the early stages while maintaining control of the diagnosis with the clinician.

### 6.2 Ethical implications and safeguard

The suggested framework does not imply any direct human experimentation and uses entirely anonymized, publicly available dataset the full scope of the ethical implications related to the use of automated diagnostic support systems in clinical settings is taken into consideration. Specifically, there are risks that these systems will be abused or overused as the sole diagnostic systems. To address this issue, the suggested CNN-GRU model will be used as a purely clinical decision-support tool, one that will help a professional to identify possible patterns of Behavior related to ASD, but not to substitute his/her judgment. The protective measures must involve upholding human-in-the-loop decision making where ultimate diagnoses must be controlled by qualified health care practitioners. Functional audits also require regular performance audits, monitoring biases, and retraining using more diverse datasets to minimize the risk of systematic errors and enhance generalization. Lastly, there is need to establish explicit clinical usage rules and ethical controls to help avoid misuse and have automated predictions used with care, especially in delicate pediatrics diagnosis cases.

### 6.3 Limitation

Although the SSBD dataset is publicly available and annotated, the systematic form of annotation, this study uses a single dataset consisting of video recordings of children acting in a predetermined Behavior sequence. Although this allows the ability to control the experimentation, it might not represent the full range of diversity and complexity of real-world manifestations of autism spectrum disorder. The Behavioral patterns of ASD may be diverse among different people, at different developmental stages, in different environmental situations, and across cultures. While we use stratified k-fold cross-validation to minimize sampling bias and to ensure that the proposed CNN GRU model is tested on similar subsets of the data, we acknowledge that the k-fold validation will not be able to offset the limited Behavioral and contextual diversity within one dataset. Such a restriction may affect the model performance when used in unconstrained real-life situations or even in the clinical environment. The same hyperparameters were used to train all architectures to create consistency and fairness of experiments. Nonetheless, various models such Efficient Net and 3D CNN-LSTM are normally prone to be optimally tuned to the architecture. This single arrangement may not wholly utilize the strengths of some of them and may rather work against them. The future work will be done to conduct systematic hyperparameter optimization specific to each model to have a more balanced and fully optimized comparison. In addition, the validation of the offered methodology on various datasets gathered across a variety of sources, such as clinical settings and home based to enhance the rigor and external validity.

## 7 Conclusion

Autism Spectrum Disorder (ASD) is a neurodevelopmental disorder that interferes with cognitive functioning, communication, and Behavioral patterns of people of various age groups. There is a strong role played by early detection of ASD toward developing a stronger learning ability and management of action in a timely manner. This study proposes a deep learning-based framework in order to analyze Behaviors associated to autism and initially distinguishing between autistic and typical Behaviors. The publicly available multiclass Self-Stimulatory Behavior Dataset (SSBD) of real-life video recordings of children with different Behavioral patterns were used as experimental data. We implemented five deep learning models CNN-GRU, MobileNet, VGG16, EfficientNet-B7, and 3DCNN-LSTM on the SSBD dataset to assessed their performance. Results show that the effectively prove the usefulness of the proposed CNN-GRU model as compared to the traditional deep learning classifiers. The findings reveal that the suggested tailored CNN-GRU model is more efficient than the other models with an accuracy of 92.94%. Such high performance presents the capability of the model in capturing both spatial and temporal patterns of Behavior in unconstrained and real life video environments. The suggested method can help experts and non-experts to recognize important Behaviors and facilitate automated Behavior monitoring application in assessing autism. In future work will be directed at the validation of the offered methodology on various datasets gathered across a variety of sources, such as naturalistic and clinical settings, to enhance the rigor and external validity.

## Data Availability

The original contributions presented in the study are included in the article/supplementary material, further inquiries can be directed to the corresponding author.
